# Effect of paternal age on clinical outcomes of *in vitro* fertilization-embryo transfer cycles

**DOI:** 10.3389/fendo.2024.1325523

**Published:** 2024-08-29

**Authors:** Xinyan Gao, Xiao Li, Fanfan Wang, Wen Cai, Shihu Sun, Shaoming Lu

**Affiliations:** ^1^ School of Clinical Medicine, Qingdao University, Qingdao, China; ^2^ Department of Obstetrics and Gynecology, The Affiliated Hospital of Qingdao University, Qingdao, Shandong, China; ^3^ Center for Reproductive Medicine, Cheeloo College of Medicine, Shandong University, Jinan, Shandong, China; ^4^ National Research Center for Assisted Reproductive Technology and Reproductive Genetics, Shandong University, Jinan, Shandong, China; ^5^ Key Laboratory of Reproductive Endocrinology of Ministry of Education, Shandong University, Jinan, Shandong, China; ^6^ Shandong Provincial Clinical Medicine Research Center for Reproductive Health, Shandong University, Jinan, Shandong, China; ^7^ Tengzhou Maternal and Child Health Hospital, Zaozhuang, Shandong, China; ^8^ Center for Reproductive Medicine, Shandong Provincial Hospital Affiliated to Shandong First Medical University, Jinan, Shandong, China

**Keywords:** paternal age, live birth rate, clinical pregnancy rate, perinatal outcomes, *in vitro* fertilization

## Abstract

**Purpose:**

This study aimed to investigate the impact of paternal age > 40 years on clinical pregnancy and perinatal outcomes among patients undergoing *in vitro* fertilization treatment.

**Methods:**

We selected 75 male patients (aged > 40 years) based on predefined inclusion and exclusion criteria. Propensity score matching was performed in a 1:3 ratio, resulting in a control group (aged ≤ 40 years) of 225 individuals. Various statistical tests, including the Mann-Whitney U test, Chi-square test, Fisher’s exact test, and binary logistic regression, were used to analyze the association between paternal age and clinical outcomes.

**Results:**

We found no statistically significant differences in semen routine parameters, clinical pregnancy outcomes, and perinatal outcomes between paternal aged > 40 and ≤ 40 years. However, in the subgroup analysis, the live birth rate significantly decreased in those aged ≥ 45 compared to those aged 41–42 and 43–44 years (31.25% vs. 69.23% and 65%, respectively; all p < 0.05). Additionally, the clinical pregnancy rate was significantly lower among those aged ≥ 45 than among those aged 41–42 (43.75% vs. 74.36%; p=0.035).

**Conclusion:**

Paternal age ≥ 45 years was associated with lower live birth and clinical pregnancy rates.

## 1 Introduction

With the implementation of China’s two- or three-child policy, an increasing number of infertile couples have resorted to assisted reproductive technology (ART) to realize their pregnancy aspirations. At the same time, the proportion of advanced couples is also increasing. It is widely acknowledged that the success rate of ART cycles diminishes with advancing female age (1). Nonetheless, there is an ongoing debate regarding the potential impact of male age on pregnancy outcomes.

Some studies suggested that males aged ≥ 40 experience a decline in sperm quality, resulting in a reduced likelihood of conception within a year (2, 3). Additionally, advanced men had a higher probability of transmitting congenital diseases, such as autism and schizophrenia, to their offspring (4, 5). Furthermore, research has demonstrated that advanced paternal age was associated with an increased risk of miscarriage, preterm birth, and lower birth weights in offspring (6).

In contrast, Chen et al. argued against the notion that advanced paternal age contributes to adverse pregnancy outcomes (7). Stern et al. found that older paternal age did not lead to an increased risk of premature delivery, low birth weight, or small gestational age among women undergoing ART treatment (8). Dain et al. suggested that paternal age might only affect fertility after age 50 (9). Moreover, certain studies have discovered that advanced paternal age had a negative impact on *in vitro* fertilization (IVF) but did not show significant effects on intracytoplasmic sperm injection (ICSI) treatments (10, 11).

This retrospective study, with a threshold of 40 years old, systematically investigated the impact of paternal age on clinical pregnancy outcomes and perinatal outcomes in couples undergoing IVF treatment.

## 2 Methods

### 2.1 Study design

This retrospective cohort study focused on infertility patients who underwent IVF treatment at the Reproductive Hospital affiliated with Shandong University between January 2015 and December 2019. A total of 2,846 patients were screened, of which 75 male patients aged > 40 were included. Propensity score matching was performed in a 1:3 ratio, resulting in the inclusion of 225 control patients aged ≤ 40. The matching criteria included maternal age, maternal body mass index (BMI), infertility duration, basic follicle-stimulating hormone (FSH) level, basic luteinizing hormone (LH) level, basic estradiol (E2) level, gonadotropin hormone-releasing hormone (GnRH) starting dose, GnRH total usage, GnRH-use days, and antral follicle count (AFC). Subgroup analysis was conducted by categorizing patients aged ≥ 40 years into three groups: 41–42, 43–44, and ≥ 45 years.

The study included patients with infertility who underwent fresh embryo transplantation, limited to the first cycle; female patients aged < 38 years with AFC > 5; and those who underwent controlled ovarian hyper-stimulation conducted using the GnRH agonist long protocol; the sole cause of infertility is tubal infertility.

The study excluded couples who utilized donor sperm; men with conditions such as azoospermia, severe asthenospermia, severe teratospermia, or severe oligospermia; men with abnormal genital findings, including prostate disease, varicocele, cryptorchidism, testicular epididymitis, testicular trauma, or surgical history; infertile women with diseases that impact the normal shape and function of the uterine cavity such as uterine endometriosis, uterine fibroids, adenomyosis, unicornuate uterus, or uterus bicornis; couples with hypertension, diabetes, or other cardiovascular diseases; and couples with chromosomal abnormalities.

### 2.2 Source of semen

All participants refrained from engaging in sexual activity for 2–7 days. Semen specimens were obtained through masturbation. The semen was ejaculated into a sterilized sputum cup that was pre-weighed and confirmed to be non-toxic to sperm. It was then labeled with the individual’s name and code. Information such as the time, color, and duration of abstinence for semen collection was recorded. The semen volume was measured, and the pH value was determined using an electronic balance. Prior to the routine semen analysis, semen liquefaction was carried out in a 37°C incubator. For the morphology analysis, sperm cells were washed following the instructions provided with the reagent kit. A 0.5 ml semen sample was then diluted in 10 ml of physiological saline. The sperm suspension underwent centrifugation at 800g for 10 minutes and was subsequently evenly spread on a glass slide using the thin smear technique. The Papanicolaou staining method was used to assess sperm morphology, and different sperm components were observed under an optical microscope. Each slide was evaluated with ≥ 200 sperm cells. All tests of the semen samples were performed in the same andrology laboratory by the same clinical operator. Finally, an interpretation of the semen analysis results was conducted. All the procedures were strictly followed according to the 5th edition of the “World Health Organization Laboratory Manual for the Examination and Processing of Human Semen” (12). Oligozoospermia was defined as three or more consecutive semen analyses showing a sperm concentration of < 15×10^6^/ml and/or a total sperm count of < 39×10^6^ per ejaculation. Asthenozoospermia was defined as progressive motility sperm (PR)% < 32% or total motility sperm (PR+NP [non-progressive motility])% < 40%. Teratozoospermia was defined as a percentage of normal-shaped sperm < 4%.

### 2.3 Controlled ovarian stimulation and embryo transfer

All infertility patients underwent a flexible long-term protocol for ovulation induction. Typically, starting from the mid-luteal phase (5–7 days after ovulation or 3–7 days of the oral contraceptive cycle), a daily injection of a short-acting GnRH agonist was administered. The dosage was 0.05–0.1 mg, and the duration of administration was 14–18 days. Throughout this period, the basic endocrine and ultrasound indicators were closely monitored. Exogenous GnRH was introduced to stimulate ovulation once the downregulation criteria were met (LH <5 IU/L, E2 <50 pg/mL, B-ultrasound: bilateral intraovarian antral follicle size <8 mm, intima <5 mm, non-functional cyst). A maintenance dose of GnRH agonist (0.100–0.033 mg) was continued until the day of human chorionic gonadotropin (HCG) withdrawal. HCG injection (4000–10000 IU) was administered based on factors such as the diameter of the 2–3 dominant follicles (reaching 20 mm), the patient’s weight, ovarian reserve, E2 level on trigger day, and number of follicles. Oocytes were retrieved within 36–38 hours after HCG injection. Corpus luteum support was initiated on the day of oocyte retrieval. Cleavage-stage embryo transfer was performed on the third day after oocyte retrieval, while blastocyst transfer occurred on the fifth day. Pregnancy confirmation took place at the first follow-up examination, which was conducted 14 days after transplantation. Subsequent follow-up procedures adhered to the hospital’s standards.

### 2.4 Outcome measures

Our study assessed multiple outcomes, including pregnancy outcomes, maternal complications, and neonatal birth weight. Specifically, we examined the rates of live birth, clinical pregnancy, preterm birth, ectopic pregnancy, and abortion. Maternal complications under investigation encompassed gestational diabetes and hypertension syndrome during pregnancy. Additionally, we analyzed neonatal outcomes such as mean birth weight (in kilograms), cases of high birth weight (> 4 kg), and cases of low birth weight (< 2.5 kg).

### 2.5 Statistical analysis

All data analyses were conducted using IBM SPSS Statistics (version 26.0; IBM, Inc) and R version 4.2.2. Categorical data are reported as counts and percentages. A chi-squared test or Fisher’s exact test was used to compare variables in these measures between the study groups. Continuous data are expressed as mean ± standard deviation and compared using the Mann-Whitney U test. All p-values were two-tailed, and a significance level of p < 0.05 was used. To investigate the association between male age and live birth rate, binary logistic regression was employed, adjusting for potential confounding factors. The adjustments included maternal age, infertility duration, semen volume, sperm concentration, sperm motility rate, normal sperm morphology rate, sperm forward motility rate, paternal BMI, testosterone, and abstinence period. Adjusted p-values, adjusted odds ratios (ORs), and 95% confidence intervals (CIs) are reported.

## 3 Results

### 3.1 Basic characteristics between the groups

The demographic data for the various study groups are presented in **Table 1**. Three hundred couples experiencing infertility were included in our study. There were no significant differences between the two groups in terms of maternal age, maternal BMI, FSH, LH, E2, GnRH starting dose, GnRH total usage, GnRH-use days, AFC, infertility duration, fertilization rate, or the number of high-quality embryos. In both groups, most patients opted to transfer the day three embryo and transfer two embryos simultaneously.

**Table 1:** Basic clinical features between the two group.

Characteristics	≤ 40 years (n=225)	>40 years (n=75)	P value
Maternal Age, years	34.22 ± 2.28	34.24 ± 2.5	0.66
Maternal BMI, kg/m^2^	23.17 ± 3.03	23.5 ± 3.14	0.29
Basic FSH, IU/L	6.87 ± 1.53	6.88 ± 1.41	0.89
Basic LH, IU/L	5.14 ± 2.53	5.24 ± 1.8	0.18
Basic E2, pg/mL	39.71 ± 35.95	41.28 ± 29.82	0.64
GnRH starting dose,IU	162.94 ± 38.47	162.33 ± 37.25	0.86
GnRH total usage,IU	1933.17 ± 751.39	1954.33 ± 707.46	0.71
GnRH use days	10.39 ± 1.98	10.47 ± 1.65	0.61
AFC	12.88 ± 4.53	13.25 ± 4.42	0.43
Infertility type,n (%)			0.93
Primary infertility rate	35(15.56)	12(15)	
Secondary infertility rate	190(84.44)	63(85)	
Infertility duration, years	3.69 ± 2.59	3.71 ± 3.05	0.44
Fertilization rate, n(%)	1493(66.53)	513(66.54)	>0.99
Number of high-quality embryos	3.9 ± 2.69	4.05 ± 2.43	0.42
Embryo transfer days			0.09
d3, n(%)	154(68.44)	59(78.67)	
d5, n(%)	71(31.56)	16(21.33)	
No. of embryos transferred, n(%)			0.03
One embryo	78(34.67)	16(21.33)	
Two embryos	147(65.33)	59(78.67)	

BMI, body mass index; FSH, follicle-stimulating hormone; LH, luteinizing hormone; E2, estradiol; GnRH, gonadotropin-releasing hormone; d3, Transfer day 3 cleavage embryos; d5, transfer day 5 blastocyst embryos; AFC, antral follicle count.

No statistically significant differences were found in the semen routine parameters between the two groups (**Table 2**). There were also no statistically significant differences in smoking, testosterone, and abstinence period between the two groups. Furthermore, subgroup analysis did not reveal any statistical differences in sperm quality parameters among the three groups (**Table 3**).

**Table 2:** Semen parameter analysis between the two group.

Variables	≤ 40 years (n=225)	>40 years (n=75)	P value
Paternal BMI, kg/m^2^	24.27 ± 3.76	25.07 ± 3.88	0.12
Semen volume, mL	3.75 ± 1.5	3.44 ± 1.49	0.11
Semen acidity, pH value	7.5 ± 0.05	7.5 ± 0.08	0.72
Sperm concentration, 10^6^/mL	61.26 ± 46	62.66 ± 40.79	0.68
Round cells concentration,10^6^/mL	0.49 ± 0.61	0.52 ± 0.54	0.42
Sperm motility rate,%	57.3 ± 19.18	55.76 ± 18.4	0.38
Sperm forward motility rate,%	44.21 ± 15.98	42.2 ± 14.8	0.36
Normal sperm morphology rate,%	5.12 ± 2.54	5.07 ± 2.9	0.78
Testosterone, ng/dl	419.68 ± 170.87	411.26 ± 209.48	0.49
Abstinence period, days	4.53 ± 2.31	4.72 ± 2	0.49
Smoking			0.54
Yes,n (%)	93(41.33)	28(37.33)	
No,n (%)	132(58.67)	47(62.67)	

**Table 3:** Subgroup analysis of semen parameters and clinical outcomes in patients >40 years old.

Outcomes	41-42 years (n=39)	43-44 years (n=20)	≥45 years (n=16)
semen volume, mL	3.43 ± 1.55	3.71 ± 1.62	3.16 ± 1.15
paternal BMI, kg/m^2^	25 ± 3.77	25.87 ± 4.82	24.22 ± 2.73
sperm concentration, 10^6^/mL	63.7 ± 34.71	47.57 ± 33.45	79 ± 56.09
sperm motility rate,%	56.6 ± 18.69	51.55 ± 17.45	58.71 ± 19.08
Sperm forward motility rate,%	42.71 ± 15.24	39.33 ± 14.14	44.39 ± 14.92
Normal sperm morphology rate,%	4.95 ± 2.63	4.61 ± 2.33	5.95 ± 3.98
Testosterone, ng/dl	414.85 ± 231.2	380.35 ± 157.62	443.15 ± 217.98
Live birth rate,n (%)	27(69.23)^a^	13(65)^b^	5(31.25)
Clinical pregnancy rate,n (%)	29(74.36)^c^	15(75)^d^	7(43.75)
Abortion rate,n (%)	2(5.13)	2(10)	2(12.5)
Gestational week, weeks	38.79 ± 1.68	38.22 ± 2.57	38.83 ± 1.38
Mean birth weight, kg	3.18 ± 0.73	3.11 ± 0.70	3.01 ± 0.49
Low birth weight rate, n (%)	5(14.71)	3(18.75)	2(28.57)

a Statistically significant differences between 41-42 years old and ≥45years old, P=0.013; ^b^Statistically significant between 43-44 years old and ≥45years old, P=0.049; ^c^ Statistically significant differences between 41-42 years old and ≥45years old, P=0.035; ^d^ Statistically significant differences between 43-44 years old and ≥45years old, adjusted P=0.035.

### 3.2 Comparison of pregnancy outcomes among groups

**Figure 1:**
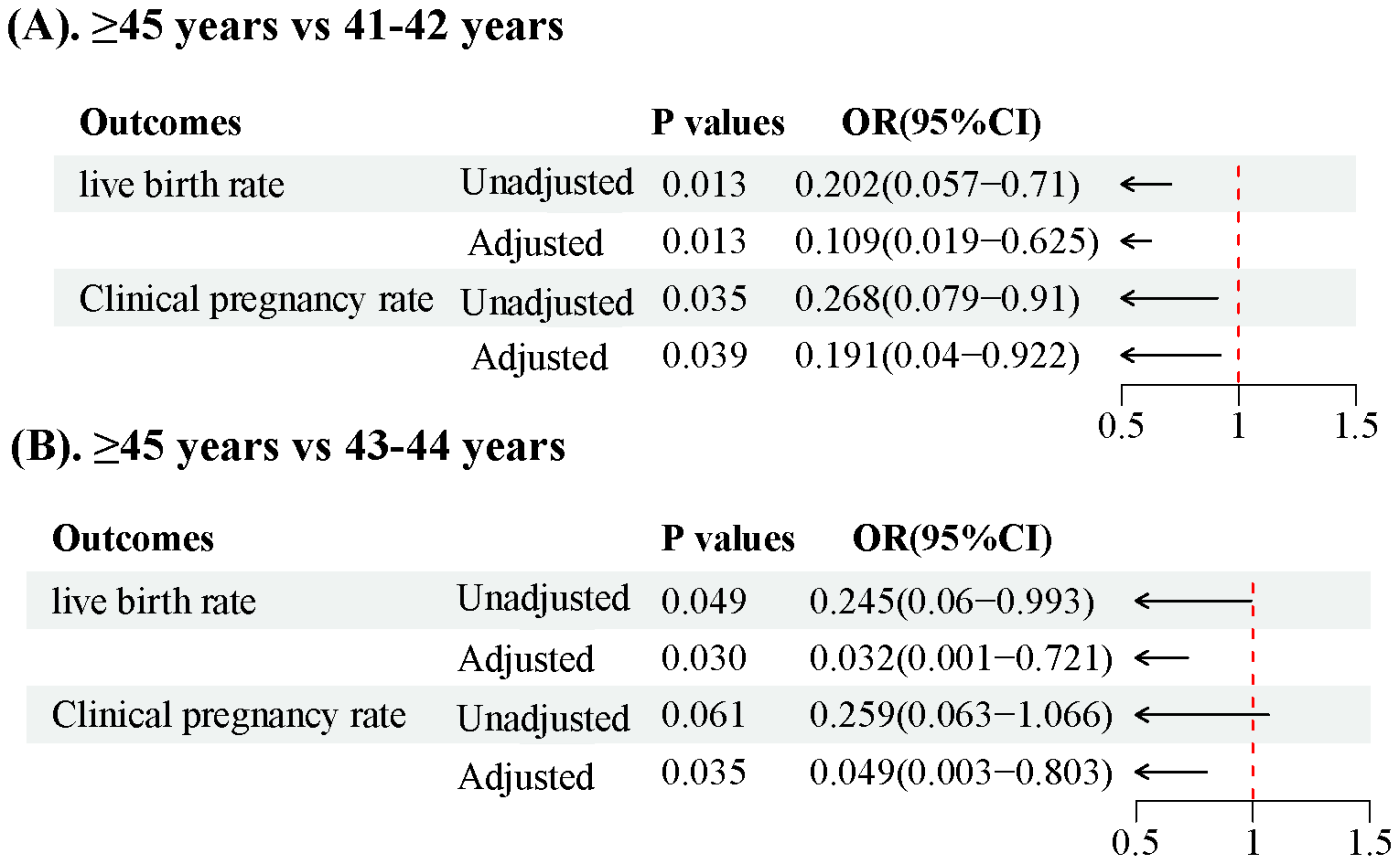
Forest plot illustrating live birth rates and clinical pregnancy rates in patients over 40 years old **(A)**. Comparison of >45 years vs 41-42 years, **(B)**. Comparison of >45 years vs 43-44 years.

We observed no statistically significant differences in the live birth, clinical pregnancy, preterm birth, ectopic pregnancy, or abortion rates (**Table 4**). However, in a subgroup analysis, a significant decrease in the live birth rate was observed in the group aged ≥ 45 years compared to those aged 41–42 years and 43–44 years (31.25% vs. 69.23% and 65%, respectively; all p < 0.05). Moreover, the clinical pregnancy rate in those aged ≥ 45 years was significantly lower than that in those aged 41–42 years(43.75% vs. 74.36%, p =0.035) (**Table 3**). We conducted adjustments for confounding factors to compare the live birth and clinical pregnancy rates between the groups aged 41–42 and ≥ 45 years and between the groups aged 43–44 and ≥ 45 years. The statistical significance of the results remained consistent (**Figure 1**).

**Table 4:** Clinical pregnancy outcome between the two group.

Outcomes	≤ 40 years (n=225)	>40 years (n=75)	P value
Live birth rate,n (%)	114(50.67)	45(60.00)	0.16
Clinical pregnancy rate,n (%)	139(61.78)	51(68.00)	0.33
Preterm birth rate,n (%)	14(6.22)	8(10.67)	0.20
Ectopic pregnancy rate,n (%)	3(2.16)	0	0.55
Abortion rate,n (%)	22(15.83)	6(11.76)	0.48

### 3.3 Comparison of perinatal complications and newborn birth weight in each group

The findings presented in **Table 5** reveal that there were no significant differences in the incidence of gestational diabetes, hypertension syndrome during pregnancy, and newborn sex compared with the control group. Additionally, no significant difference was noted in gestational weeks between the two groups. Moreover, the two groups showed no significant differences in the average birth weight, rate of high birth weight, and rate of low birth weight. The results from the subgroup analysis in **Table 3** also showed no significant difference.

**Table 5:** Pregnancy complications and newborn birth weight between the two group.

Outcomes	≤ 40 years (n=225)	>40 years (n=75)	P value
Gestational week, weeks	38.6 ± 2.1	38.59 ± 1.92	0.90
Gestational diabetes, n (%)	9(4)	3(4)	>0.99
Hypertension syndrome during pregnancy, n (%)	6(2.7)	3(4)	0.70
Newborn sex, n (%)			0.96
Male	78(54.55)	32(54.24)	
Female	65(45.45)	27(45.76)	
Mean birth weight, kg	3.16 ± 0.67	3.14 ± 0.68	0.87
High birth weight rate, n (%)	16(11.19)	4(6.78)	0.44
Low birth weight rate, n (%)	23(16.08)	10(16.95)	0.84

## 4 Discussion

We conducted a retrospective study to explore the impact of paternal age on pregnancy outcomes in patients undergoing IVF, analyzing 5 years of clinical data. We observed a significant decrease in live birth and clinical pregnancy rates when men were aged ≥ 45 years. These results suggest that pregnancy outcomes were adversely affected as male age increased. We observed that in the ≤40 age group, 68.44% opted for D3 transfer while 31.56% chose D5 transfer. In the >40 age group, 78.67% preferred D3 transfer and 21.33% selected D5 transfer. Although a higher proportion in both groups favored D3 transfer, there was no statistically significant difference between the two groups. This implies that the day of transfer does not affect the conclusions of this study. It is worth noting that according to existing literature, blastocyst transfer leads to improved pregnancy outcomes (13, 14). In future studies, we intend to conduct more comprehensive research on the influence of embryo transfer day on pregnancy outcomes in older patients.

Currently, it is widely believed that semen parameters decrease with increasing age in men. However, there is still controversy regarding which parameters decrease and at which age range this decline begins. Kidd et al. conducted a study comparing patients aged 30 and 50, which revealed that 50-year-old patients showed a significant decline in semen volume, sperm motility, and normal sperm morphology rates. However, there were no significant differences in sperm concentration (15). Stone et al. conducted another study that found semen parameters to be relatively stable until the age of 34, but a decrease in both sperm concentration and normal form rate was observed after age 40. Additionally, a decline in sperm motility and the forward sperm movement rate was observed after age 43, and ejaculation volume showed a decrease after age 45 (16). Dain et al. performed a comprehensive review of studies and found a linear decrease in semen volume with paternal age. Although there is no correlation between male age and sperm concentration, motility, and morphology, the total sperm count and motility decrease due to the reduced semen volume (9). There are several reasons for the decline in semen parameters in elderly men. These include 1) decreased testicular function, leading to a decreased number of interstitial cells, supporting cells, and germ cells, as well as decreased testosterone levels; 2) a decline in accessory gland function, where inadequate seminal vesicle function reduces semen volume and prostate atrophy reduces the water and protein content in ejaculation, resulting in reduced ejaculation volume and sperm vitality; 3) cellular and physiological changes, such as a decrease in antioxidant capacity, reduced ability to repair cell and tissue damage, leading to abnormal sperm morphology; and 4) structural changes in male reproductive anatomy, including narrowing of the convoluted seminiferous tubules, vascular dysfunction, and age-related systemic diseases (15, 17–22). Our study observed a declining trend in semen parameters among male patients aged > 40. These parameters included semen volume, semen concentration, sperm motility, normal sperm morphology rate, and forward motility rate. However, we did not find any significant difference between the two groups. This observation suggests that a significant decline in semen parameters might only occur in men over 50 (15, 23, 24). However, it is also possible that during the initial screening of male patients, we excluded those with common male diseases such as varicocele and prostate diseases in order to further reduce confounding factors caused by male diseases. The incidence of these diseases increases with age, and by excluding them, the sperm quality was indirectly improved. Multicenter, prospective studies with larger sample sizes should be performed in the future to confirm these results. Factors such as gonadal infections, smoking, alcohol abuse, and other unhealthy lifestyle habits can also contribute to decreased sperm quality. The pH value and round cell concentration of the semen in both groups remained within the normal range, reducing the confounding effects of gonadal infections in the patients.

Several studies have examined the impact of paternal age on clinical pregnancy rates. It has been suggested that advanced paternal age negatively affects the clinical pregnancy rate in patients with oligospermia but has no significant impact when sperm concentration is normal (25). Soares et al. conducted a retrospective study on the use of donor eggs for infertility treatment and observed a decrease in clinical pregnancy rates in recipients aged > 45 (26). Similarly, Gallardo et al. investigated donor cycles for infertility treatment and found comparable rates of fertilization and clinical pregnancy regardless of male age (27). Consistent with these findings, our research reveals a decline in the clinical pregnancy rate in men aged > 45. Therefore, advanced male age could have detrimental effects on clinical pregnancy outcomes.

Live birth rates are influenced by the age of the couple experiencing infertility, as indicated by several studies. For women aged 38–40, some studies have shown a 50% decrease in live birth rates, with an additional 50% decrease occurring at age 40 (2). According to Klonoff-Cohen et al., the live birth rate is estimated to be 38% for men aged 35, 17% for men aged 36–40, and 7% for men over 40. Moreover, the likelihood of a failed live birth increases by 12% each year as paternal age advances (10). However, certain articles argue against a correlation between paternal age and live birth rate during ICSI (28, 29). Consequently, controversy still exists regarding the impact of male age on live birth rates. Our study observed no significant difference in live birth rates when comparing the two groups using the age 40 threshold. Nevertheless, when we raised the threshold to 45, we found a significant decrease in live birth rates among men aged ≥ 45. This phenomenon may be attributed to the higher likelihood of older men transmitting genetically inferior material, leading to adverse pregnancy outcomes (3). Hence, advanced male age has been shown to reduce live birth rates, particularly for men aged > 45.

Some studies have indicated a correlation between increased paternal age and higher miscarriage rates among patients undergoing artificial insemination. This relationship may be attributed to the potential impact of paternal age on genomic integrity and subsequent embryo development (30–32). For instance, a study conducted in France found a miscarriage rate of 32.4% for fathers aged ≥ 45, while fathers aged < 30 had a miscarriage rate of 13.7% (33). If the female partner is below the age of 38, the oocyte can repair potential DNA damage in the sperm during fertilization or later stages of embryo development (34). No significant negative impact of advanced age in males on the miscarriage rate was observed in our study. This may be attributed to the fact that all female partners included in our research were under the age of 38. It is likely that the influence of female factors on the rate of miscarriage is more evident. If the female partner is also of advanced age, the decline in oocyte quality exceeds the scope of repair, leading to a decrease in live birth rates and an elevation in miscarriage rates (35, 36).

Alio et al. reported a 19% increase in the incidence of low birth weight in neonates when comparing fathers aged > 45 to those aged 25–29 years (37). Similarly, Reichman et al. (38) conducted a cohort study and found that fathers aged ≥ 35 had a higher risk of neonatal low birth weight than those aged 20–34. On the contrary, a Japanese study (39) found a positive correlation between paternal age and the incidence of low birth weight. In our study, although we did not observe a significant difference in newborn birth weight, We found a decreasing trend in birth weight when comparing fathers aged ≥ 45 to those aged 41–42 (3.01 ± 0.49 vs. 3.18 ± 0.73). It is worth noting that the newborn’s birth weight is influenced by various factors, including the mother’s nutritional status, for which we did not have detailed information. Consequently, we were unable to control for the influence of maternal factors on newborn birth weight. Future follow-up studies should employ more stringent criteria to investigate the possible correlation between advanced paternal age and low birth weight in newborns.

This study is a retrospective, single-center analysis with its inherent limitations. In addition, our hospital was unable to collect comprehensive information on factors such as maternal weight and nutritional status throughout the pregnancy, thereby lacking reliable data to elucidate the impact of paternal age on newborns. Lastly, due to the strict inclusion criteria, a relatively small number of patients aged ≥45 years were included in our sample. In the future, multicenter studies with larger sample sizes are needed to validate our results.

## 5 Conclusion

The increase in paternal age could lead to adverse pregnancy outcomes. For patients with infertility, clinicians should pay attention to the impact of advanced paternal age on adverse pregnancy outcomes and advocate for men to complete their reproductive plans before age 45.

## Data Availability

The original contributions presented in the study are included in the article/supplementary material. Further inquiries can be directed to the corresponding author.
